# A location-based serious game in museum learning: a comparative field study of visitor experience, motivation, cognitive load, and objective learning outcomes

**DOI:** 10.3389/fpsyg.2026.1979842

**Published:** 2026-10-30

**Authors:** Qirui Zhang, Xinyu Cao, Yu Hou

**Affiliations:** 1 College of Art, Anhui Xinhua University, Hefei, Anhui, China; 2 College of Design, Hanyang University, Seoul, Republic of Korea

**Keywords:** audio guide, cognitive load, cultural heritage, location-based serious game, motivation, museum learning, visitor experience

## Abstract

**Introduction:**

This study compared three museum visiting modalities—natural visits, audio guides, and a location-based serious game—in relation to visitor experience, motivation, cognitive load, and objective learning outcomes. Although serious games have increasingly been used in cultural heritage and museum education, less is known about how location-based serious games perform when compared with common visiting modalities in authentic museum settings.

**Methods:**

A mixed-methods non-randomized comparative field study was conducted at Chengdu Museum with 105 adult visitors (35 participants in each condition). Data were collected through post-visit questionnaires and a 10-item exhibition-based knowledge test. A subset of 17 participants took part in semi-structured interviews.

**Results:**

Participants in the serious game condition reported significantly higher engagement, perceived knowledge/learning, meaningful experience, and emotional connection than those in the natural visit and audio guide conditions. They also achieved higher objective knowledge-test scores. However, the motivational advantage of the serious game was limited to the comparison with the audio guide condition, while the cognitive-load components showed distinct patterns across conditions. Intrinsic cognitive load was higher in both the audio guide and serious game conditions than in natural visiting, extraneous cognitive load was lower in the serious game condition than in both comparison conditions, and germane cognitive load was higher in the serious game condition than in both comparison conditions. Given the limited internal consistency of these measures, the cognitive-load findings were interpreted as exploratory. Interview findings further indicated that narrative tasks, exhibit-based clues, spatial movement, and feedback were associated with active exploration and closer observation, while also involving additional task-related mental effort.

**Discussion:**

These findings suggest that location-based serious games may support more active, meaningful, and learning-oriented museum experiences, while their motivational benefits and cognitive demands may vary across outcome domains.

## 1 Introduction

Museums are increasingly understood not only as institutions for preserving and displaying objects, but also as experience-oriented environments that support informal learning, cultural interpretation, and visitor participation. In contemporary museum learning, visitors are not simply passive recipients of exhibition information; they construct meaning through observation, movement, emotional response, cognitive processing, and social interaction (Falk and Dierking, 2013; Packer and Ballantyne, 2016). This shift has encouraged museums to adopt digital and interactive technologies, including mobile guides, augmented reality, mixed reality, and game-based systems, to support more active forms of cultural heritage learning (Pallud, 2017; Trunfio et al., 2022; Zhou et al., 2022).

Serious games have attracted growing attention in museum and cultural heritage contexts because they combine educational purposes with rules, tasks, narrative structures, feedback, and situated interaction. Unlike simple gamification, which adds isolated game elements to existing activities, serious games organize learning through more complete game-based systems involving goals, challenges, decision-making, progression, and feedback (Kapp, 2012; Nicholson, 2012). In museum settings, these mechanisms can transform exhibition content into interactive learning tasks, encouraging visitors to observe artifacts more carefully, interpret cultural information more actively, and connect historical narratives with personal and social meanings (Bellotti et al., 2013; Mortara et al., 2014).

Among different forms of museum serious games, location-based serious games are especially relevant to on-site museum learning. In this study, a location-based serious game refers to a museum game in which narrative tasks, clue discovery, and learning activities are organized around visitors' physical movement, exhibit locations, gallery routes, and on-site interaction. In such games, museum space is not merely a setting for play; it becomes part of the learning process itself. From a situated learning perspective, learning is shaped by interaction with specific contexts and activities (Brown et al., 1989). This spatially situated feature distinguishes location-based serious games from screen-based cultural heritage games or virtual learning applications that are separated from physical museum environments (Nofal et al., 2020; Othman et al., 2021; Stanković Elesini et al., 2022).

However, the educational value of museum serious games should not be assumed to be uniformly positive. Previous research suggests that interactive technologies and serious games may support engagement, motivation, and knowledge acquisition, but they may also introduce additional cognitive demands (Boyle et al., 2016; Connolly et al., 2012; Xu et al., 2023). Self-determination theory provides a complementary motivational perspective, suggesting that choice, challenge, and feedback may support sustained engagement and motivation (Ryan and Deci, 2000). Because autonomy, competence, and relatedness were not directly measured in this study, this perspective is used for theoretical interpretation rather than as a tested need-based model. In location-based museum games, visitors may need to understand rules, operate a mobile interface, follow routes, search for clues, complete tasks, and process exhibition information at the same time. From the perspective of cognitive load theory, learning may be hindered when task-related demands exceed visitors' available cognitive resources (Sweller, 1988; Sweller et al., 2011). Cognitive load can further be distinguished into intrinsic, extraneous, and germane components, which reflect different forms of cognitive demand during learning (Sweller et al., 2011; Leppink et al., 2013). Therefore, evaluating museum serious games requires attention not only to experiential and motivational benefits, but also to the cognitive effort required during the visit.

Visitor experience, motivation, cognitive-load components, and objective knowledge performance provide complementary perspectives for evaluating museum learning. Visitor experience is particularly important because museum visits are voluntary, self-directed, affective, and situated within specific spatial and cultural contexts. In this study, visitor experience is conceptualized through four dimensions of the Museum Experience Scale: engagement, perceived knowledge/learning, meaningful experience, and emotional connection (Othman et al., 2011). Motivation reflects visitors' willingness to participate, sustain attention, and invest effort, while intrinsic, extraneous, and germane cognitive load reflect different forms of cognitive demand. Objective knowledge performance provides an immediate post-visit measure based on exhibition content, with perceived knowledge/learning and interview data used as complementary evidence for interpreting visitors' learning experiences.

Despite growing interest in museum technologies and cultural heritage games, several gaps remain. First, many studies have evaluated digital museum technologies or cultural heritage games in isolation, without directly comparing them with common museum visiting modalities. Second, fewer studies have examined experiential, motivational, and cognitive responses within the same research design. Third, field-based evidence from authentic museum settings, especially cultural heritage museums in China, remains limited. Natural visits, audio guides, and location-based serious games represent different configurations of visitor agency, interpretive structure, spatial behavior, and learning activity. Comparing these three modalities can therefore provide a clearer understanding of how different forms of museum interaction are associated with visitor experience, motivation, cognitive-load components, and objective knowledge performance (Othman et al., 2011; Pallud, 2017; Tang and Zhou, 2025).

To address these gaps, the present study conducted a mixed-methods comparative field study at Chengdu Museum. The study compared three visiting conditions: natural visit, audio guide, and a location-based serious game. It examined four domains of visitor response: visitor experience, motivation, cognitive-load components, and objective knowledge performance. Visitor experience was assessed through engagement, perceived knowledge/learning, meaningful experience, and emotional connection. Motivation and cognitive-load components were examined as psychological and cognitive response variables, while objective knowledge performance was assessed using a knowledge test based on exhibition content. Semi-structured interviews were also used to interpret visitors' perceptions of the narrative, task-based, exhibit-based, and location-based interaction mechanisms of the serious game condition.

This study contributes to museum learning and serious game research in three ways. First, it examines a location-based serious game in an authentic museum field setting rather than in a laboratory or purely virtual environment. Second, it compares three common visiting modalities—natural visits, audio guides, and a location-based serious game—rather than evaluating a single digital intervention in isolation. Third, it integrates experiential, motivational, cognitive, and objective knowledge indicators to provide a more balanced account of how location-based serious games may support museum learning while also introducing additional cognitive demands.

Based on the literature and research gaps discussed above, the present study proposes a theoretically informed comparative framework. Visiting modality was treated as the grouping variable, while visitor experience, motivation, cognitive-load components, and objective knowledge performance were examined as key response variables. Visitor experience included four dimensions: engagement, perceived knowledge/learning, meaningful experience, and emotional connection. Situated learning, motivation, and cognitive load provide complementary perspectives for interpreting these relationships. The framework also examined the associations among visitor experience, motivation, cognitive-load components, and objective knowledge performance.

As shown in Figure 1, the framework compares visitor responses across the three visiting modalities and examines associations among the main study variables. The proposed relationships are theoretically informed and do not represent tested causal or mediational pathways.

**Figure 1:**
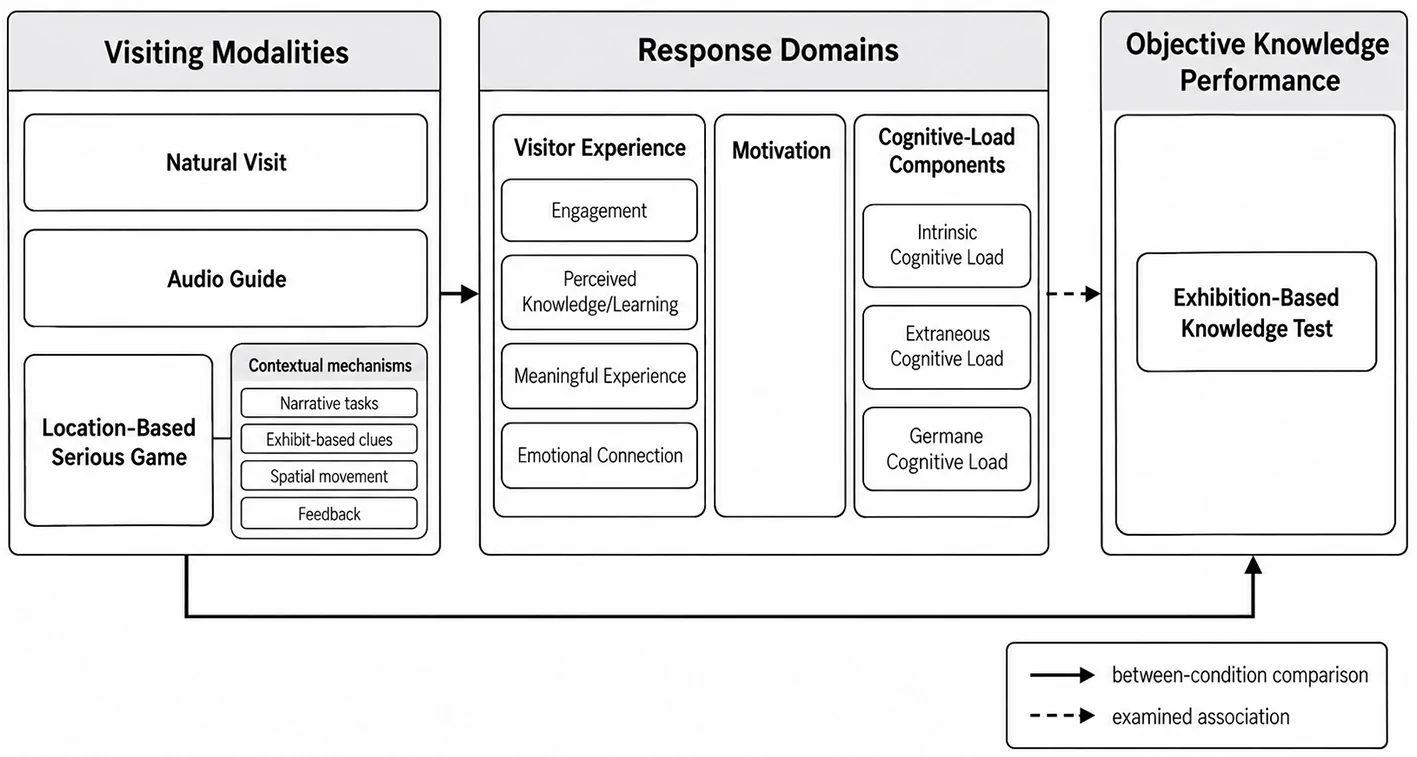
Theoretically informed framework for comparing the three museum visiting modalities. Connections represent theoretically informed relationships rather than tested causal pathways.

Accordingly, the present study addresses the following research questions:

RQ1. How do visitor experiences differ across natural visit, audio guide, and location-based serious game conditions?
RQ2. How do motivation and cognitive-load components differ across the three visiting modalities?
RQ3. Does objective knowledge performance differ across natural visit, audio guide, and serious game conditions?
RQ4. How are visitor experience, motivation, cognitive-load components, and objective knowledge performance associated with one another?
RQ5. How do visitors perceive the narrative, task-based, exhibit-based, and location-based interaction mechanisms of the serious game?

Based on these research questions, the following hypotheses were developed:

H1. Participants in the serious game condition will report higher visitor experience scores than those in the natural visit and audio guide conditions.
H1a. Participants in the serious game condition will report higher engagement than those in the natural visit and audio guide conditions.
H1b. Participants in the serious game condition will report higher perceived knowledge/learning than those in the natural visit and audio guide conditions.
H1c. Participants in the serious game condition will report higher meaningful experience than those in the natural visit and audio guide conditions.
H1d. Participants in the serious game condition will report higher emotional connection than those in the natural visit and audio guide conditions.
H2. Participants in the serious game condition will achieve higher objective knowledge performance than those in the natural visit and audio guide conditions.
H3. Participants in the serious game condition will report higher motivation than those in the natural visit and audio guide conditions.
H4. Visitor experience and motivation will be positively associated with objective knowledge performance.
H5. Cognitive-load components will differ among the natural visit, audio guide, and serious game conditions. Associations between the cognitive-load components and objective knowledge performance will be examined exploratorily.

## 2 Materials and methods

### 2.1 Research design

This study adopted a mixed-methods comparative field design to examine differences among three museum visiting modalities: natural visit, audio guide, and a location-based serious game (Creswell and Plano Clark, 2018). The study was conducted in an authentic museum setting and focused on visitor experience, motivation, cognitive load, and objective learning outcomes. Because participants were not randomly assigned to the three conditions, the study is more appropriately characterized as a non-randomized comparative field study rather than a randomized controlled experiment. This design preserved the ecological validity of an authentic museum setting while providing less control over pre-existing individual differences among participants (Shadish et al., 2002).

The quantitative component compared post-visit questionnaire responses and exhibition-based knowledge-test scores across the three conditions and examined associations among the main study variables. The qualitative component consisted of semi-structured interviews that provided contextual evidence for interpreting visitors' perceptions of the serious game condition, particularly its narrative tasks, exhibit-based clues, spatial movement, and feedback mechanisms. Accordingly, between-condition differences are interpreted as associations rather than definitive causal effects of visiting modality.

### 2.2 Research setting and participants

The study was conducted at Chengdu Museum, a comprehensive city museum in Southwest China. The empirical setting was Exhibition Hall 2, which presents the historical and cultural development of Chengdu from the Pre-Qin period to the Han, Wei, Jin, and Northern and Southern Dynasties. This exhibition area was selected because it provided a common content base across the three visiting conditions and sufficient historical and cultural material for assessing visitor experience and immediate post-visit knowledge performance.

A total of 105 adult museum visitors were included in the quantitative analyses, with 35 participants in each condition. Participants ranged from 18 to 44 years of age (*M* = 26.33, *SD* = 6.46), and the sample comprised 45 male and 60 female participants. Only complete and valid responses were retained in the final quantitative dataset.

Age and sex were available for all participants and were examined as background characteristics across conditions. Educational background, prior museum experience, prior knowledge, interest in history or cultural heritage, and previous digital-game experience were not systematically available in the archived dataset and therefore could not be retrospectively included as covariates.

For the qualitative component, a subset of 17 participants took part in semi-structured interviews after the visit. The interviews focused on visitors' experience, task understanding, exhibit observation, perceived learning, motivation, and cognitive effort. Particular attention was given to participants' perceptions of the narrative, task-based, exhibit-based, and location-based interaction mechanisms of the serious game condition.

No formal a priori power analysis was conducted before data collection. A sensitivity analysis based on the achieved sample indicated that, with *N* = 105, three groups, α = 0.05, and power = 0.80, the study could detect an omnibus effect of approximately Cohen's *f* = 0.31 (η^2^ ≈ 0.086). The sample therefore provided reasonable sensitivity to moderate and larger effects but comparatively limited sensitivity to small effects.

### 2.3 Visiting conditions and serious game design

Three visiting conditions were compared: natural visit, audio guide, and location-based serious game. All conditions were organized around the same exhibition area in Chengdu Museum to maintain comparability across groups.

The natural visit condition served as the baseline modality. Participants explored the exhibition freely according to their own interests, route choices, prior knowledge, and available time. They did not receive systematic digital guidance, audio interpretation, or game-based tasks. Their visits were mainly shaped by the exhibition layout, exhibit labels, visual observation, and personal interpretation.

The audio guide condition represented a structured interpretive modality. Participants received curated audio explanations related to selected exhibits and historical-cultural content. The audio guide provided a relatively linear interpretive structure and helped visitors connect individual artifacts with broader exhibition narratives. This condition therefore represented a guided form of museum learning based primarily on listening and information reception.

The location-based serious game condition represented a situated interactive modality. Participants experienced the exhibition through a mobile game-based visiting activity embedded in the physical museum space. The game included narrative tasks, exhibit-based clues, spatial movement, task completion, and feedback. Participants followed game prompts, moved through the exhibition area, identified relevant artifacts or clues, completed task-related responses, and proceeded according to the feedback provided by the game.

The serious game was examined as an on-site museum learning modality rather than as a screen-based application detached from the exhibition space. Its core design features included narrative framing, task-based progression, location-based navigation, exhibit-based clues, and feedback. Through these features, the game encouraged visitors to engage with the exhibition through observation, searching, comparison, and interpretation.

Figure 2 illustrates representative interface screenshots and exhibit-based tasks from the location-based serious game.

**Figure 2:**
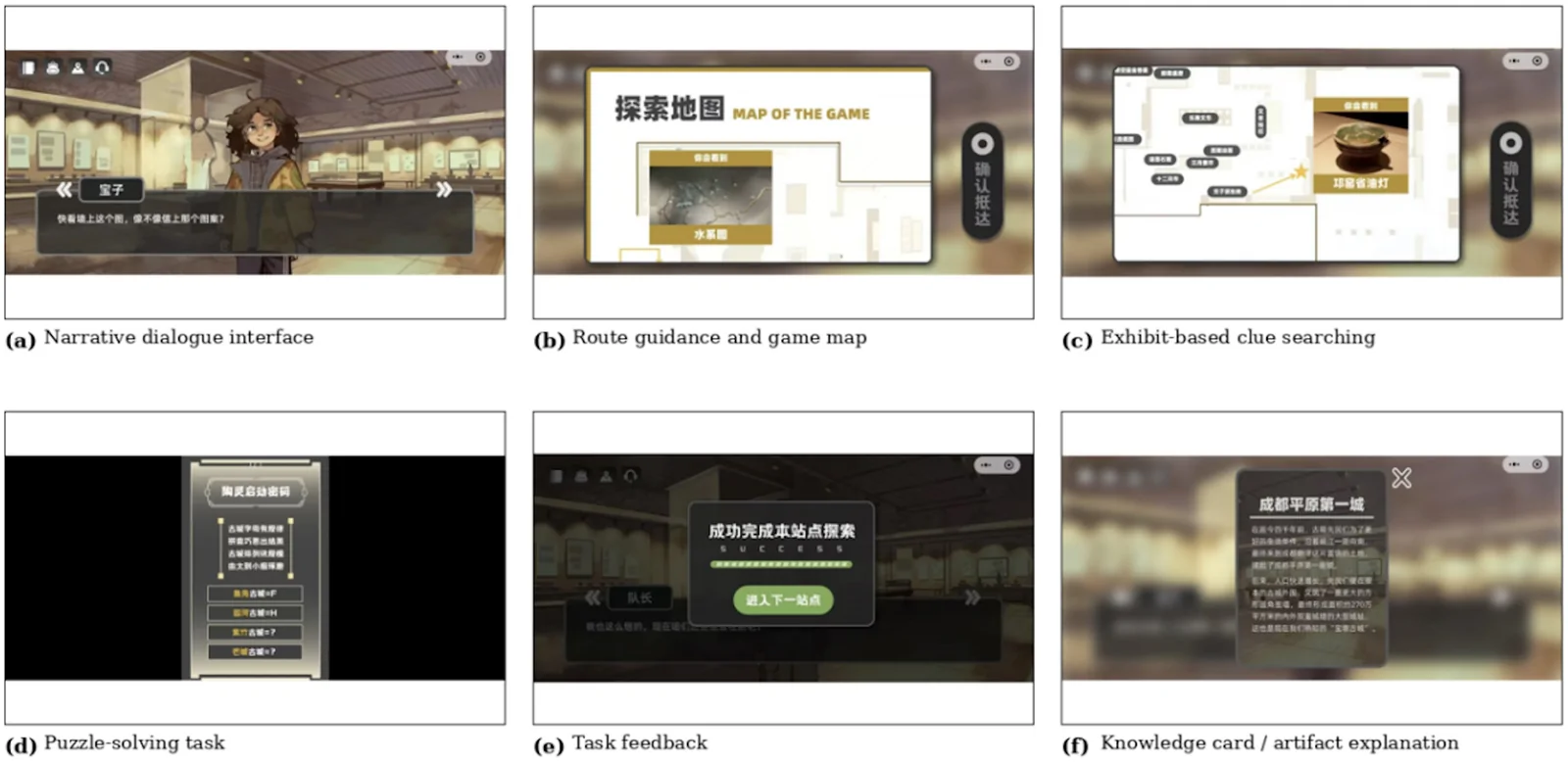
Interface screenshots and exhibit-based tasks of the location-based serious game. Representative screenshots illustrate the game interface, narrative tasks, exhibit-based clues, and task progression used in the location-based serious game condition. **(a)** Narrative dialogue interface. **(b)** Route guidance and game map. **(c)** Exhibit-based clue searching. **(d)** Puzzle-solving task. **(e)** Task feedback. **(f)** Knowledge card / artifact explanation. Game interface screenshots from 博物请归位 (Bowu Qing Gui Wei), offered at Chengdu Museum, China. Reproduced with permission from the game team; screenshots captured and compiled by the authors.

### 2.4 Measures

Data were collected through a post-visit questionnaire, an exhibition-based knowledge test, and semi-structured interviews. The questionnaire and knowledge test were administered in Chinese, with English translations provided in the Supplementary material.

The post-visit questionnaire measured visitor experience, motivation, and cognitive load. It contained 34 Likert-type items. Visitor experience was measured through four dimensions of the Museum Experience Scale: engagement, perceived knowledge/learning, meaningful experience, and emotional connection (Othman et al., 2011). Each dimension contained five items. Engagement referred to visitors' attention and involvement during the visit. Perceived knowledge/learning referred to visitors' perceived understanding of exhibition content. Meaningful experience captured the personal or cultural significance attributed to the visit. Emotional connection reflected visitors' affective responses to exhibits, historical narratives, and cultural content.

Motivation was measured with six items reflecting intrinsic and extrinsic motivational tendencies during the visit. Because the two three-item subsets showed limited internal consistency, the six items were retained as a composite motivation measure and interpreted cautiously.

Cognitive load was measured with eight items representing intrinsic cognitive load (two items), extraneous cognitive load (three items), and germane cognitive load (three items) (Sweller et al., 2011; Leppink et al., 2013). These components were analyzed separately rather than combined into a single cognitive-load score. Because of their limited internal consistency, the component-level findings were treated as exploratory.

Objective knowledge performance was assessed through a 10-item exhibition-based knowledge test.

Five items focused on Chengdu's history and culture during the Pre-Qin period, and five items focused on content related to the Han, Wei, Jin, and Northern and Southern Dynasties. Each correct answer received one point, and each incorrect answer received zero points. Total scores ranged from 0 to 10, with higher scores indicating better objective knowledge performance.

The semi-structured interviews addressed visitors' overall experience, task comprehension, exhibit observation, perceived learning, motivation, cognitive effort, and responses to the serious game's narrative, task-based, exhibit-based, and location-based interaction mechanisms. Questions were asked flexibly according to participants' responses and visiting conditions, and the interview guide is provided in the Supplementary material. The interview data were used for contextual interpretation rather than statistical inference.

### 2.5 Procedure

Data collection was conducted on site at Chengdu Museum. Participants first received information about the study procedures and provided written informed consent. They then completed one of the three visiting modalities: natural visit, audio guide, or location-based serious game. Participants were allocated to the three conditions according to a predetermined rotating sequence; individual randomization was not used. All three conditions were implemented within the same exhibition area and were based on the same core exhibition content to improve comparability across conditions.

After the visit, participants completed the post-visit questionnaire and the exhibition-based knowledge test. A subset of participants subsequently took part in semi-structured interviews focusing on visiting experience, exhibit understanding, task participation, perceived learning, motivation, and cognitive effort.

Figure 3 summarizes the field study procedure. The procedure consisted of seven stages: participant recruitment and study briefing, participation in one of the three visiting conditions, completion of the museum visit, post-visit questionnaire, exhibition-based knowledge test, semi-structured interviews, and data analysis.

**Figure 3:**
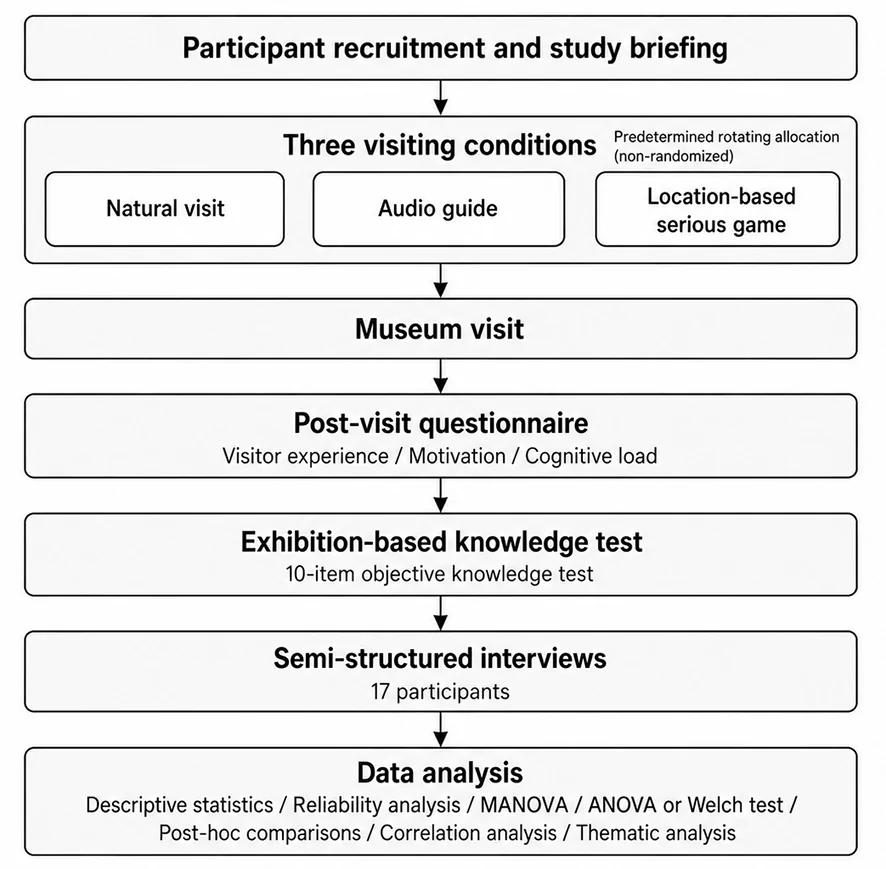
Procedure of the field study. The procedure included participant recruitment and study briefing, participation in one of the three visiting conditions, completion of the museum visit, post-visit questionnaire, exhibition-based knowledge test, semi-structured interviews, and data analysis.

### 2.6 Data analysis

Quantitative data were analyzed using descriptive statistics, reliability analysis, multivariate and univariate group comparisons, *post-hoc* tests, and correlation analysis. Means, standard deviations, and 95% confidence intervals were calculated for the main study variables.

An initial multivariate analysis of variance (MANOVA) was conducted across the eight questionnaire outcomes using Pillai's trace. Significant multivariate effects were followed by one-way ANOVA with Tukey HSD comparisons when homogeneity of variance was satisfied, or Welch's ANOVA with Games-Howell comparisons when this assumption was violated. Holm correction was applied across the outcome-level tests, and effect sizes were reported as η^2^.

Objective knowledge performance was analyzed separately using the same variance-based procedure. Given the non-randomized design, supplementary regression analyses adjusting for age and sex were conducted using HC3 robust standard errors.

Pearson correlations were used to examine associations among the main study variables, with Benjamini-Hochberg false discovery rate correction applied across the correlation matrix. Statistical significance was set at p < 0.05. Quantitative analyses were conducted using Python 3.13.5 with SciPy 1.17.0 and statsmodels 0.14.6.

Interview data were analyzed using thematic analysis (Braun and Clarke, 2006). Responses were documented through written field notes rather than verbatim transcripts and were expanded into interview summaries immediately after each interview. Two researchers reviewed and coded the summaries, compared their interpretations, and resolved differences through consensus. Related codes were then grouped into broader themes.

## 3 Results

### 3.1 Participant characteristics, descriptive statistics, and reliability

The final dataset included 105 valid participants, with 35 participants in each visiting condition. Mean age was 27.91 years (*SD* = 7.16) in the natural visit condition, 26.74 years (*SD* = 6.76) in the audio guide condition, and 24.34 years (*SD* = 4.88) in the serious game condition, with no significant between-group difference, *F*_(2, 102)_ = 2.88, *p* = 0.061. Sex distribution differed significantly across conditions, χ^2^ (2, *N* = 105) = 7.23, *p* = 0.027. Because the study used a non-randomized design, age and sex were considered in supplementary sensitivity analyses.

Scale scores were calculated by averaging the corresponding item-level responses for each questionnaire dimension. The objective knowledge-test score was calculated as the total number of correct answers out of ten.

Internal consistency was examined before the main analyses. As shown in Table 1, engagement and perceived knowledge/learning showed Cronbach's α values of 0.805 and 0.782, respectively, while meaningful experience and emotional connection showed α values of 0.679 and 0.677. Motivation showed lower internal consistency (α = 0.609). For the cognitive-load components, the Spearman–Brown coefficient was 0.368 for intrinsic cognitive load, and Cronbach's α values were 0.523 for extraneous cognitive load and 0.644 for germane cognitive load. Accordingly, findings related to motivation and cognitive load were interpreted with caution.

**Table 1:** Internal consistency of the questionnaire measures.

Measure	Number of items	Reliability coefficient
Intrinsic cognitive load	2	Spearman–Brown = 0.368
Extraneous cognitive load	3	α = 0.523
Germane cognitive load	3	α = 0.644
Motivation	6	α = 0.609
Perceived knowledge/learning	5	α = 0.782
Engagement	5	α = 0.805
Meaningful experience	5	α = 0.679
Emotional connection	5	α = 0.677

The Spearman–Brown coefficient is reported for the two-item intrinsic cognitive-load measure; Cronbach's α is reported for the remaining measures.

The objective knowledge test was scored separately and was not included in the reliability analysis of the Likert-type questionnaire measures.

Descriptive statistics for the main study variables are presented in Table 2. Across the four visitor experience dimensions, the serious game condition showed the highest mean scores for engagement, perceived knowledge/learning, meaningful experience, and emotional connection. The natural visit and audio guide conditions showed relatively similar descriptive patterns across these dimensions.

**Table 2:** Descriptive statistics across the three visiting conditions.

Variable	NV M (SD), 95% CI	AG M (SD), 95% CI	SG M (SD), 95% CI
Engagement	3.98 (0.62), [3.77, 4.20]	4.02 (0.49), [3.85, 4.19]	4.62 (0.26), [4.53, 4.71]
Perceived knowledge/learning	3.97 (0.51), [3.80, 4.15]	4.07 (0.41), [3.93, 4.21]	4.62 (0.31), [4.51, 4.72]
Meaningful experience	3.93 (0.57), [3.74, 4.13]	4.05 (0.43), [3.90, 4.19]	4.51 (0.34), [4.40, 4.63]
Emotional connection	3.73 (0.62), [3.51, 3.94]	3.86 (0.43), [3.71, 4.01]	4.30 (0.38), [4.17, 4.43]
Motivation	4.06 (0.49), [3.89, 4.23]	3.95 (0.39), [3.82, 4.08]	4.21 (0.37), [4.08, 4.34]
Intrinsic cognitive load	2.31 (0.62), [2.10, 2.53]	2.71 (0.50), [2.54, 2.89]	2.97 (0.82), [2.69, 3.25]
Extraneous cognitive load	2.26 (0.62), [2.04, 2.47]	2.21 (0.51), [2.03, 2.39]	1.90 (0.48), [1.73, 2.06]
Germane cognitive load	3.57 (0.57), [3.38, 3.77]	3.79 (0.49), [3.62, 3.96]	4.29 (0.48), [4.12, 4.45]
Objective knowledge performance	6.60 (1.88), [5.95, 7.25]	7.11 (1.66), [6.54, 7.68]	8.03 (1.07), [7.66, 8.40]

NV, natural visit; AG, audio guide; SG, serious game; CI, confidence interval.

Figure 4 illustrates the mean scores of the four visitor experience dimensions across the three visiting conditions. Consistent with the descriptive statistics, the serious game condition showed the highest scores across all four dimensions.

**Figure 4:**
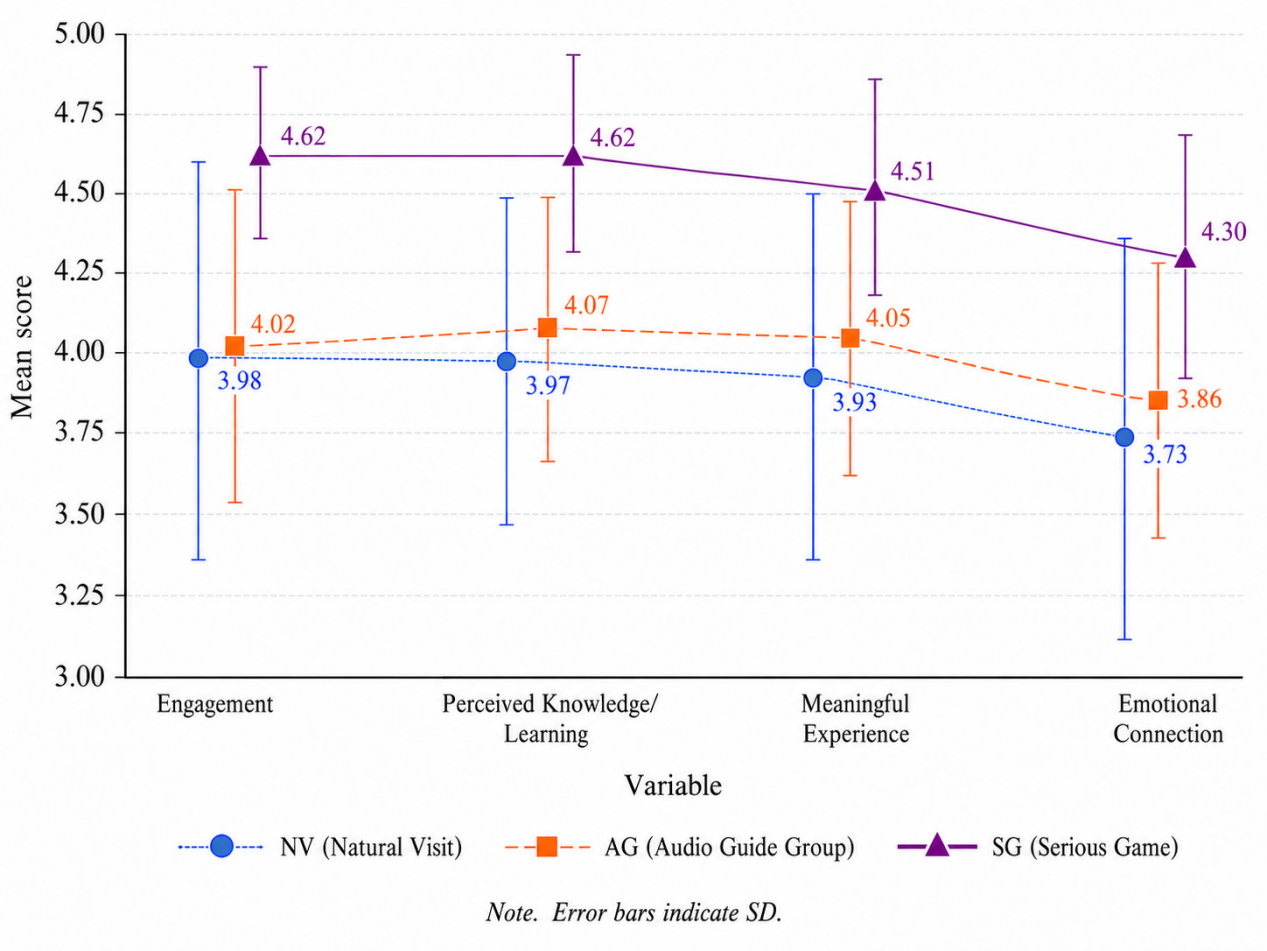
Visitor experience dimensions by group. Mean scores are shown for engagement, perceived knowledge/learning, meaningful experience, and emotional connection across the natural visit, audio guide, and serious game conditions.

Motivation scores were relatively high across all three conditions, with the serious game condition showing the highest mean score. The cognitive-load components showed distinct descriptive patterns across conditions: intrinsic cognitive load was highest in the serious game condition, extraneous cognitive load was lowest in the serious game condition, and germane cognitive load was highest in the serious game condition.

Figure 5 presents the mean scores for motivation and the three cognitive-load components across the three visiting conditions.

**Figure 5:**
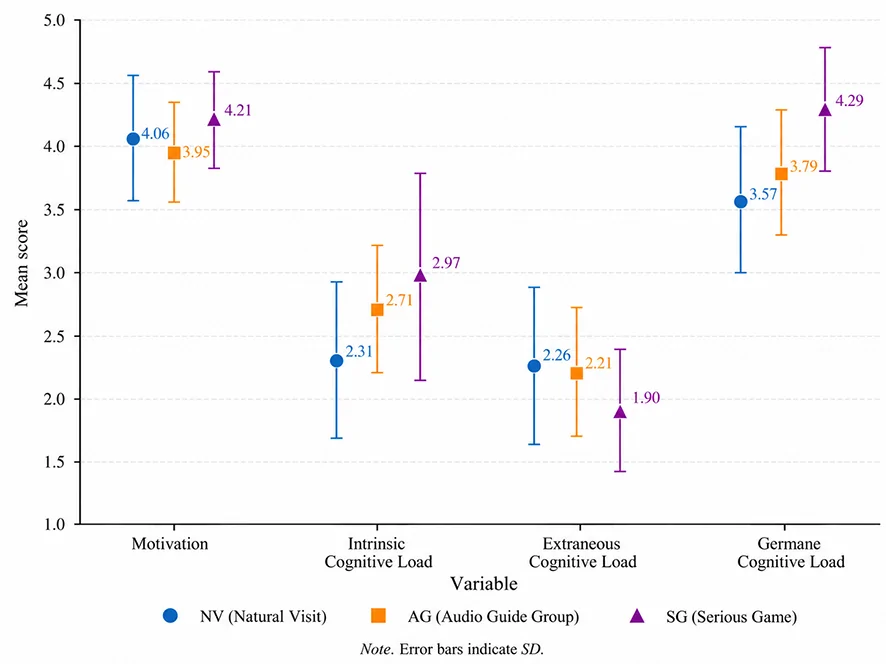
Motivation and cognitive-load components by group. Mean scores are shown for motivation, intrinsic cognitive load, extraneous cognitive load, and germane cognitive load across the natural visit, audio guide, and serious game conditions.

For objective knowledge performance, the serious game condition achieved the highest mean score, followed by the audio guide and natural visit conditions.

Figure 6 shows the distribution of objective knowledge-test scores across the three visiting conditions.

**Figure 6:**
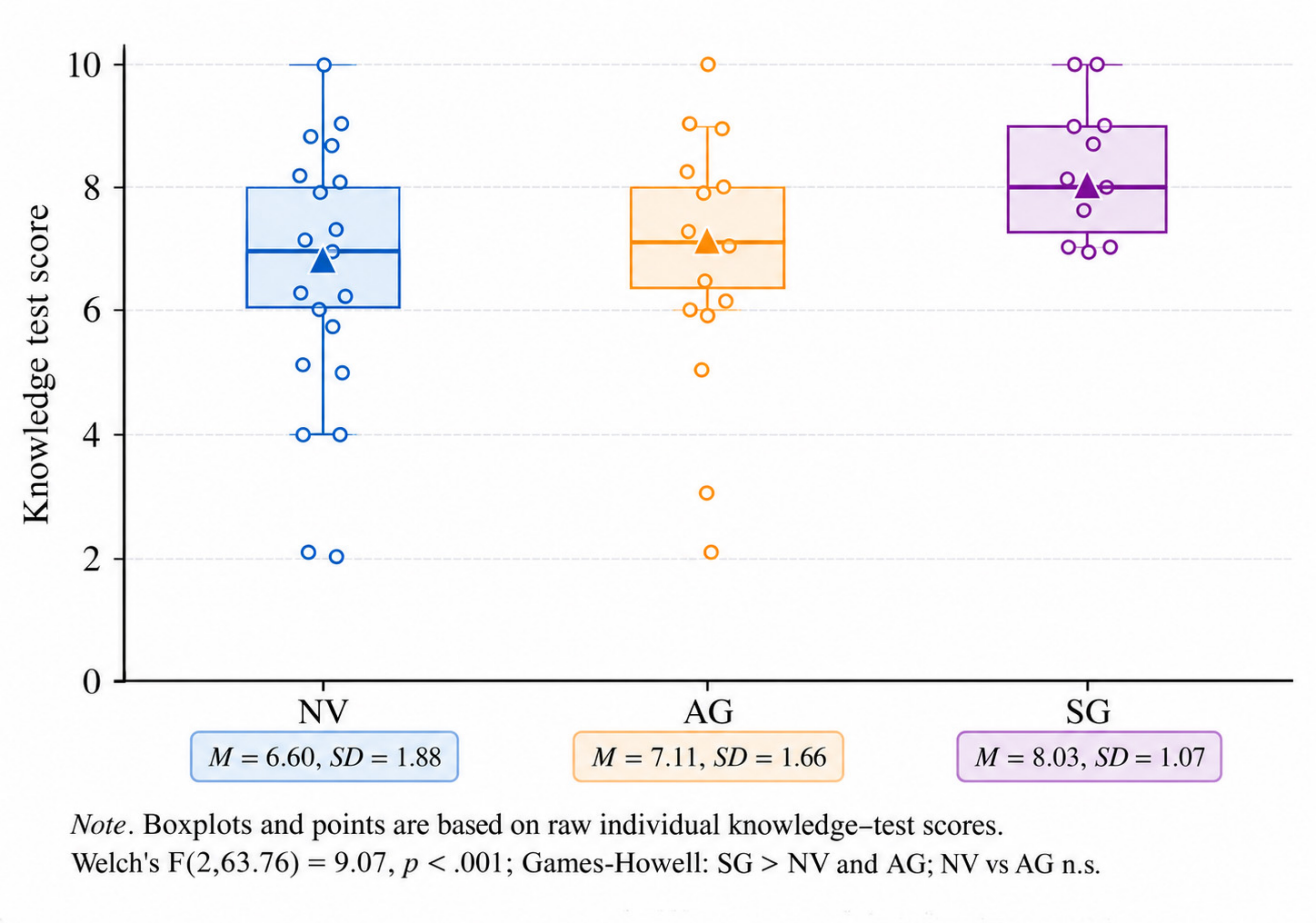
Objective knowledge performance by group. Scores are shown for the 10-item exhibition-based knowledge test across the natural visit, audio guide, and serious game conditions. Boxplots and points are based on raw individual knowledge-test scores. Welch's F_(2, 63.76)_ = 9.07, *p* < 0. 001; Games-Howell: SG > NV and AG; NV vs AG n.s.

Overall, the descriptive results showed higher visitor-experience scores and objective knowledge performance in the serious game condition. Motivation showed comparatively smaller descriptive differences, while the cognitive-load components displayed distinct patterns across conditions.

### 3.2 Group differences across visiting conditions

An initial MANOVA across the eight questionnaire outcomes showed a significant overall effect of visiting condition, Pillai's trace = 0.583, *F*_(16, 192)_ = 4.94, *p* < 0.001. Follow-up univariate analyses were conducted with Holm correction across the outcome-level tests. The results are summarized in Table 3.

**Table 3:** Group comparison results across the three visiting conditions.

Variable	Levene *p*	Analysis	Test result	Holm-adjusted p	η^2^	*Post-hoc* conclusion
Engagement	0.016	Welch + Games-Howell	*F*_(2, 59.47)_ = 30.26	< 0.001	0.275	SG > NV and AG; NV vs. AG n.s.
Perceived knowledge/learning	0.143	ANOVA + Tukey HSD	*F*_(2, 102)_ = 24.32	< 0.001	0.323	SG > NV and AG; NV vs. AG n.s.
Meaningful experience	0.014	Welch + Games-Howell	*F*_(2, 65.22)_ = 19.79	< 0.001	0.238	SG > NV and AG; NV vs. AG n.s.
Emotional connection	0.008	Welch + Games-Howell	*F*_(2, 65.89)_ = 15.87	< 0.001	0.209	SG > NV and AG; NV vs. AG n.s.
Motivation	0.200	ANOVA + Tukey HSD	*F*_(2, 102)_ = 3.41	0.037	0.063	SG > AG; other comparisons n.s.
Intrinsic cognitive load	0.173	ANOVA + Tukey HSD	*F*_(2, 102)_ = 8.77	0.001	0.147	AG > NV; SG > NV; SG vs. AG n.s.
Extraneous cognitive load	0.325	ANOVA + Tukey HSD	*F*_(2, 102)_ = 4.64	0.024	0.083	SG < NV and AG; NV vs. AG n.s.
Germane cognitive load	0.193	ANOVA + Tukey HSD	*F*_(2, 102)_ = 17.66	< 0.001	0.257	SG > NV and AG; NV vs. AG n.s.
Objective knowledge performance	0.046	Welch + Games-Howell	*F*_(2, 63.76)_ = 9.07	0.001	0.127	SG > NV and AG; NV vs. AG n.s.

NV, natural visit; AG, audio guide; SG, serious game; ns, not significant. η^2^ values are reported as effect-size estimates for group differences. Holm correction was applied across the outcome-level tests, and Tukey HSD or Games-Howell adjustment was used for pairwise comparisons within each outcome.

Significant group differences were found across all four visitor experience dimensions. Participants in the serious game condition reported higher engagement, perceived knowledge/learning, meaningful experience, and emotional connection than those in both comparison conditions (all adjusted *p* < 0.001; η^2^ = 0.209–0.323). The natural visit and audio guide conditions did not differ significantly.

For motivation, a significant group effect was observed, *F*_(2, 102)_ = 3.41, adjusted *p* = 0.037, η^2^ = 0.063. The serious game condition showed higher motivation than the audio guide condition, whereas the other pairwise comparisons were not significant.

The cognitive-load components showed different patterns. Intrinsic cognitive load was higher in the audio guide and serious game conditions than in natural visiting, *F*_(2, 102)_ = 8.77, adjusted *p* = 0.001, η^2^ = 0.147. Extraneous cognitive load was lower in the serious game condition than in both comparison conditions, *F*_(2, 102)_ = 4.64, adjusted *p* = 0.024, η^2^ = 0.083. Germane cognitive load was higher in the serious game condition than in both comparison conditions, *F*_(2, 102_) = 17.66, adjusted *p* < 0.001, η^2^ = 0.257. Given the limited internal consistency of these measures, these findings were interpreted as exploratory.

For objective knowledge performance, Welch's ANOVA showed a significant group difference, Welch's *F*_(2, 63.76)_ = 9.07, adjusted *p* = 0.001, η^2^ = 0.127. The serious game condition achieved higher scores than both the natural visit and audio guide conditions, whereas the two comparison conditions did not differ significantly.

Supplementary analyses adjusting for age and sex yielded the same substantive conclusions for the principal visitor-experience, motivation, and objective knowledge outcomes.

Figure 7 summarizes the effect-size estimates for the group differences. The largest effects were observed for perceived knowledge/learning, engagement, and germane cognitive load, whereas motivation and extraneous cognitive load showed comparatively smaller effects.

**Figure 7:**
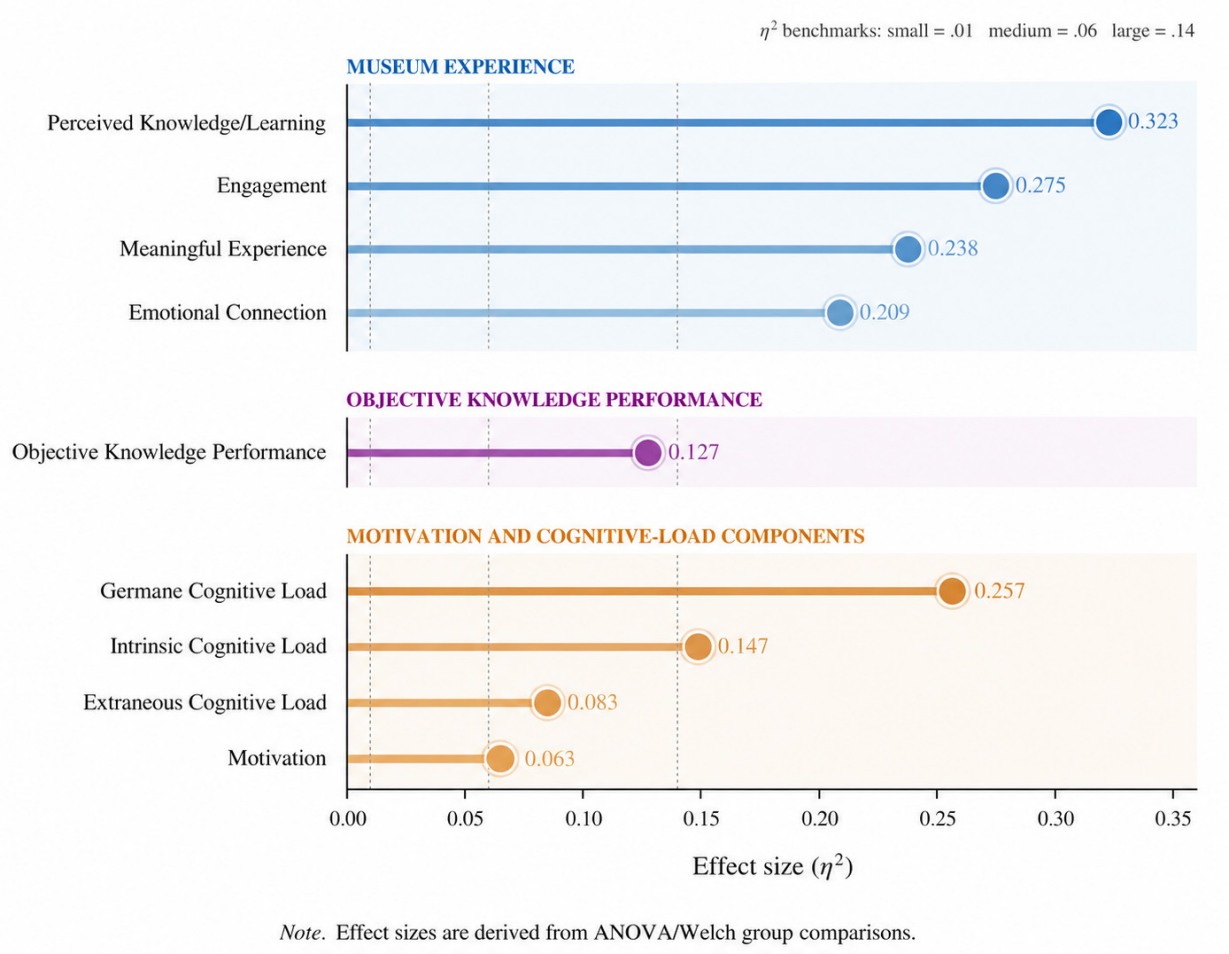
Effect-size estimates for group differences across study variables. The figure summarizes η^2^ values for the four visitor experience dimensions, motivation, the three cognitive-load components, and objective knowledge performance. Effect sizes are derived from ANOVA/Welch group comparisons.

Taken together, the group comparison results indicate that the serious game condition was associated with higher visitor experience scores and stronger objective knowledge performance than both natural visiting and audio guidance. The motivational difference was comparatively limited, while the three cognitive-load components showed distinct patterns across conditions.

### 3.3 Associations among main study variables

Pearson correlations were examined among the main study variables, with Benjamini-Hochberg false discovery rate correction applied across the correlation matrix. The results are presented in Figure 8.

**Figure 8:**
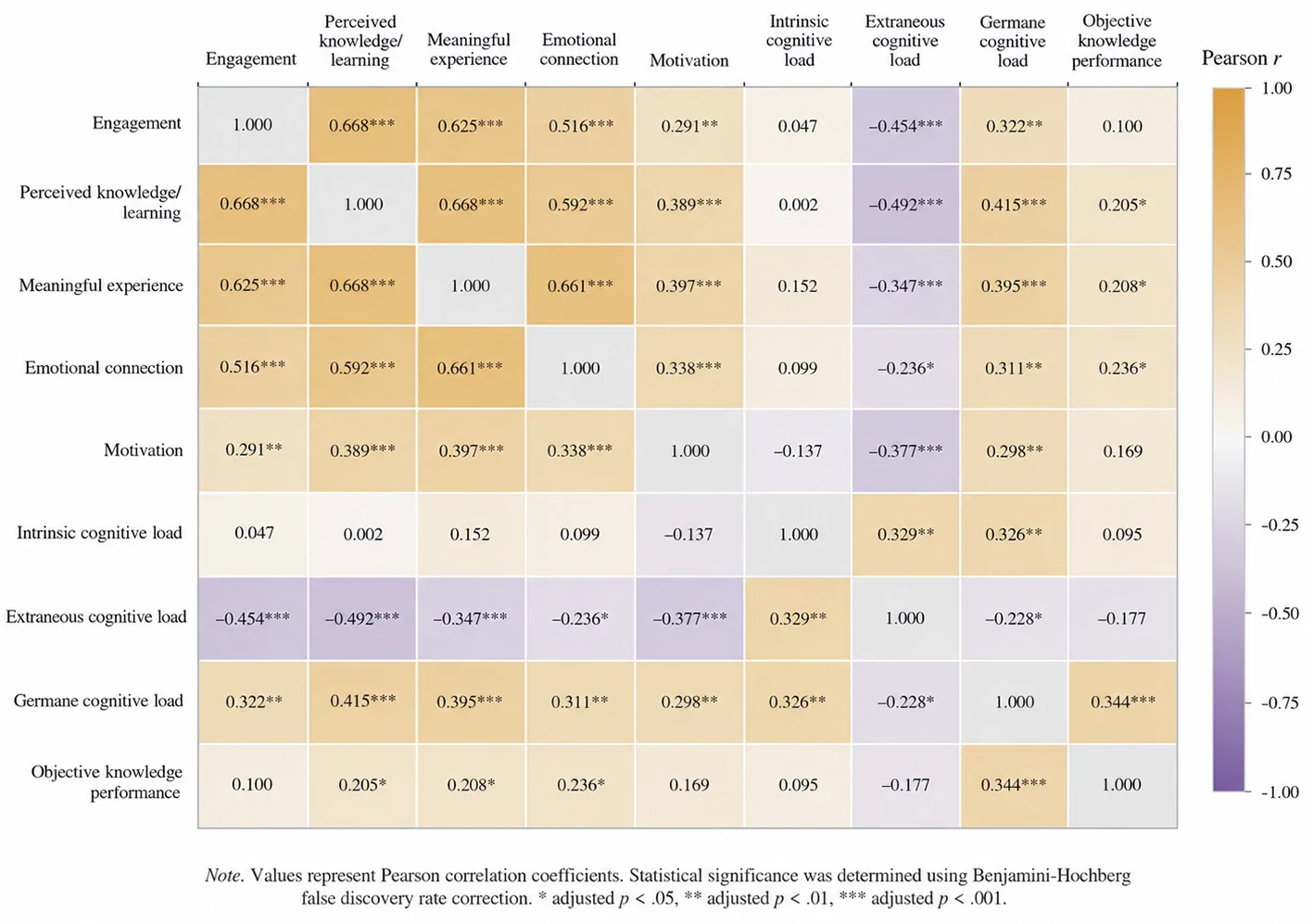
Correlation matrix of the main study variables. Values represent Pearson correlation coefficients among the nine study variables. Statistical significance was determined using Benjamini-Hochberg false discovery rate correction. *adjusted *p* < 0.05, **adjusted *p* < 0.01, ***adjusted *p* < 0.001.

Figure 8 presents the correlation matrix of the main study variables.

The four visitor experience dimensions were positively correlated with one another (*r* = 0.516–0.668, all adjusted *p* < 0.001). Motivation was also positively associated with all four visitor experience dimensions (*r* =0.291–0.397, all adjusted *p* < 0.01).

Objective knowledge performance showed weak positive associations with perceived knowledge/learning (*r* = 0.205, adjusted *p* = 0.048), meaningful experience (*r* = 0.208, adjusted p = 0.046), and emotional connection (*r* = 0.236, adjusted *p* = 0.023). Germane cognitive load was also positively associated with objective knowledge performance (*r* = 0.344, adjusted *p* < 0.001). Engagement, motivation, intrinsic cognitive load, and extraneous cognitive load were not significantly associated with objective knowledge performance.

Given the limited reliability of the cognitive-load components, these associations were interpreted cautiously. The correlation results indicate associations rather than causal relationships.

### 3.4 Qualitative findings

The qualitative interview data were used to provide contextual evidence for interpreting the quantitative results. Based on the thematic analysis of the interview summaries, five main themes were identified: narrative-guided exploration, exhibit-based reasoning, emotional connection with artifacts, increased task demand, and active visiting experience. These themes are summarized in Table 4.

**Table 4:** Summary of qualitative interview themes.

Theme	Description	Related findings
Narrative-guided exploration	Participants described the storyline as helping them follow the exhibition route and focus on selected exhibits.	Engagement; meaningful experience
Exhibit-based reasoning	Game tasks encouraged visitors to search for clues, observe details, compare information, and connect exhibit information.	Perceived knowledge/learning; objective knowledge performance
Emotional connection with artifacts	Some participants reported stronger curiosity or emotional responses toward artifacts through game-based interaction.	Emotional connection
Increased task demand	Some participants felt that clue searching, puzzle solving, route following, and mobile interaction required additional attention and effort.	Cognitive-load components
Active visiting experience	Participants described the game-based visit as more active and participatory than ordinary viewing or listening-based interpretation.	Overall visitor experience

The first theme was narrative-guided exploration. Participants described the storyline as helping them follow the exhibition route and focus on selected exhibits. In comparison with ordinary viewing, the narrative structure provided a clearer sense of progression and helped participants understand how individual exhibits were connected to broader historical and cultural contexts. This theme corresponded mainly to the higher engagement and meaningful experience scores observed in the serious game condition.

The second theme was exhibit-based reasoning. Participants reported that the game tasks encouraged them to search for clues, observe exhibit details, compare information, and make task-related judgments based on the exhibition content. This finding suggests that the serious game condition encouraged closer object-centered observation rather than only general browsing. The theme was consistent with the higher perceived knowledge/learning scores and stronger objective knowledge-test performance in the serious game condition.

The third theme was emotional connection with artifacts. Some participants indicated that the game-based tasks increased their curiosity about specific artifacts and made the exhibition content feel more vivid and personally meaningful. By linking tasks with objects and historical narratives, the serious game appeared to support affective responses to the exhibition. This theme provided contextual evidence for the higher emotional connection scores reported by participants in the serious game condition.

The fourth theme was increased task demand. While participants generally described the serious game as engaging, some also noted that clue searching, puzzle solving, route following, and mobile interaction required additional attention and effort. This theme provided contextual evidence for the distinct cognitive-load patterns observed in the quantitative analyses and indicated that game-based interaction could involve additional task-related mental effort.

The fifth theme was active visiting experience. Participants described the game-based visit as more participatory than ordinary viewing or listening-based interpretation. The tasks, clues, and feedback encouraged them to interact with the exhibition more actively and to treat the visit as a process of exploration and problem solving. This theme helped explain why the serious game condition showed stronger results across the visitor experience dimensions.

Overall, the qualitative findings suggest that the serious game experience was characterized by narrative progression, exhibit-based tasks, spatial exploration, and feedback. Participants described these features as supporting active observation, meaning-making, and emotional engagement, while also involving additional task-related effort.

### 3.5 Summary of hypothesis testing

The hypothesis-testing results are summarized in Table 5. H1 and its four sub-hypotheses were supported. Participants in the serious game condition reported significantly higher visitor experience scores than those in both the natural visit and audio guide conditions across the four experience dimensions: engagement, perceived knowledge/learning, meaningful experience, and emotional connection.

**Table 5:** Summary of hypothesis testing results.

Hypothesis	Result	Evidence
H1	Supported	SG > NV and AG across the four visitor experience dimensions.
H1a	Supported	Engagement was significantly higher in SG than in NV and AG.
H1b	Supported	Perceived knowledge/learning was significantly higher in SG than in NV and AG.
H1c	Supported	Meaningful experience was significantly higher in SG than in NV and AG.
H1d	Supported	Emotional connection was significantly higher in SG than in NV and AG.
H2	Supported	Objective knowledge-test scores were significantly higher in SG than in NV and AG.
H3	Partially supported	Motivation was significantly higher in SG than in AG, but SG did not differ significantly from NV.
H4	Partially supported	Objective knowledge performance was positively associated with perceived knowledge/learning, meaningful experience, and emotional connection, but not with engagement or motivation.
H5	Supported	Intrinsic cognitive load was higher in AG and SG than in NV; extraneous cognitive load was lower in SG than in NV and AG; and germane cognitive load was higher in SG than in NV and AG. Germane cognitive load was positively associated with objective knowledge performance, whereas intrinsic and extraneous cognitive load were not.

NV, natural visit; AG, audio guide; SG, serious game.

H2 was supported. Participants in the serious game condition achieved significantly higher objective knowledge performance than those in both comparison conditions. H3 was partially supported, as the serious game condition showed higher motivation than the audio guide condition but did not differ significantly from natural visiting.

H4 was partially supported. Objective knowledge performance was positively associated with perceived knowledge/learning, meaningful experience, and emotional connection, but not with engagement or motivation. H5 was supported for between-condition differences in the cognitive-load components. Exploratory correlations further showed that only germane cognitive load was positively associated with objective knowledge performance.

Taken together, the results indicate that the location-based serious game condition was associated with stronger visitor experience and objective knowledge performance than natural visiting and audio guidance. The motivational difference was comparatively limited, while the cognitive-load components showed distinct patterns across conditions and were interpreted cautiously because of their limited internal consistency.

## 4 Discussion

### 4.1 Main findings

Overall, the findings indicate that the location-based serious game condition was associated with stronger visitor experience and objective knowledge performance than natural visiting and audio guidance. This pattern was most evident in the four dimensions of visitor experience: engagement, perceived knowledge/learning, meaningful experience, and emotional connection. At the same time, the results also show that the advantages of the serious game condition were not uniform across all outcome variables. Its motivational advantage was limited to the comparison with the audio guide condition, while intrinsic, extraneous, and germane cognitive load showed distinct patterns across conditions.

These findings suggest that the serious game provided a more structured and participatory form of museum visiting. Compared with natural visiting, it offered clearer tasks and interpretive direction. Compared with audio guidance, it required visitors to engage more actively with exhibits rather than only receive explanatory information. The combination of storyline, exhibit-based clues, spatial movement, and feedback was associated with closer observation and more purposeful engagement with exhibition content.

The cognitive-load findings add an important qualification to this interpretation. Intrinsic cognitive load was higher in both the audio guide and serious game conditions than in natural visiting, whereas extraneous cognitive load was lower and germane cognitive load was higher in the serious game condition than in both comparison conditions. These distinct patterns indicate that the serious game did not uniformly increase cognitive demand across all components. Given the limited internal consistency of the cognitive-load measures, however, these findings should be interpreted as exploratory.

The correlation results further clarify the relationship between experience and learning. Objective knowledge performance was modestly associated with perceived knowledge/learning, meaningful experience, and emotional connection, but not with engagement or motivation. Among the cognitive-load components, germane cognitive load was positively associated with objective knowledge performance, whereas intrinsic and extraneous cognitive load were not. Given the limited reliability of these measures, these associations should be interpreted cautiously. This pattern suggests that objective knowledge performance in museum learning may depend less on general activity or interest alone and more on whether visitors develop meaningful and emotionally grounded connections with exhibition content.

The qualitative findings support this interpretation by showing how participants experienced the serious game as an active and guided form of visiting. The storyline helped organize the route, the tasks directed attention to selected exhibits, and the clues encouraged object-based reasoning. At the same time, participants also recognized that game-based visiting required additional attention and effort. These findings suggest that the serious game functioned not only as an engagement tool, but also as an interpretive structure that shaped how visitors observed, selected, and processed exhibition information.

### 4.2 Theoretical implications

This study contributes to museum learning research by showing that different visiting modalities are associated with different configurations of experience, cognitive effort, and learning performance. Museum learning is not simply a matter of information exposure, but is shaped by how visitors move through the exhibition, direct their attention, interact with artifacts, and connect exhibition content with personal or cultural meaning. This interpretation is consistent with situated learning, which emphasizes the role of context and activity in shaping learning processes (Brown et al., 1989).

The findings also contribute to serious game research in cultural heritage contexts. The value of the serious game condition did not appear to derive simply from the addition of game elements, but from organizing museum visiting as a task-based and exhibit-centered process involving narrative, clue searching, spatial movement, and feedback. Self-determination theory provides a complementary perspective for interpreting the observed motivational and engagement patterns, although autonomy, competence, and relatedness were not directly measured and therefore cannot be treated as tested mechanisms (Ryan and Deci, 2000).

The findings further highlight the importance of distinguishing among cognitive-load components rather than treating cognitive load as a single construct. The differing patterns of intrinsic, extraneous, and germane cognitive load suggest that location-based serious games may involve qualitatively different forms of cognitive demand. In particular, the positive association between germane cognitive load and objective knowledge performance is consistent with the possibility that some cognitive effort accompanies active processing of exhibition content. However, given the limited reliability of the component measures and the non-randomized design, these patterns should be interpreted as exploratory rather than causal.

The results also refine the role of engagement in informal museum learning. Engagement was closely related to other experiential dimensions but was not significantly associated with objective knowledge performance. By contrast, perceived knowledge/learning, meaningful experience, and emotional connection showed modest positive associations with objective knowledge performance. This pattern suggests that attention and participation alone may be insufficient; their educational value may depend on whether they are connected to meaningful interpretation, emotional relevance, and exhibit-based understanding.

### 4.3 Design implications

The findings suggest that museum serious games should be designed as interpretation tools rather than entertainment layers. Their main value lies in guiding visitors' attention, movement, observation, and reasoning in ways that support meaningful engagement with exhibition content.

First, narrative should be closely connected to the exhibition structure. A storyline can help visitors understand why specific exhibits matter and how individual objects relate to broader historical or cultural themes. Narrative design should therefore serve interpretation rather than operate as a detached fictional frame.

Second, tasks should require exhibit-based reasoning. Game activities are more likely to support learning when they direct visitors to observe artifact details, compare information, identify clues, and make judgments based on exhibition content. Tasks that can be completed without careful attention to the exhibits may increase participation but are less likely to support knowledge acquisition.

Third, location-based interaction should do more than move visitors from one point to another. Route design should help visitors understand relationships among objects, galleries, chronological sequences, and cultural narratives. Spatial movement becomes educationally meaningful when it supports interpretation rather than simple navigation.

Fourth, emotional connection should be treated as part of the learning design. The positive association between emotional connection and objective knowledge performance suggests that affective engagement may be relevant to immediate knowledge performance. Serious games can support this connection through role setting, narrative tension, artifact-centered tasks, and feedback that links cultural objects with human stories and historical contexts.

Finally, cognitive-load components should be actively considered in design. Museum serious games should minimize unnecessary extraneous demands while maintaining appropriate task challenge and supporting germane processing. Excessive task difficulty, unclear clues, dense instructions, or complex interface operations may distract visitors from the exhibits. Designers should therefore keep instructions concise, link clues directly to artifacts, provide feedback that supports understanding, and ensure that mobile interaction remains secondary to object observation.

### 4.4 Limitations and future research

Several limitations should be acknowledged. First, the study was conducted in one exhibition area of Chengdu Museum. Although this authentic field setting strengthened ecological validity, the findings may not be generalizable to other museum types, exhibition themes, visitor groups, or cultural contexts. Future research should examine location-based serious games in different museum and heritage environments.

Second, the study adopted a non-randomized comparative field design. Although participants were allocated according to a predetermined rotating sequence, individual randomization was not used, which limited control over pre-existing participant differences. Age did not differ significantly across conditions, whereas sex distribution differed significantly. In addition, educational background, prior museum experience, prior knowledge, interest in history or cultural heritage, and previous digital-game experience were not systematically available for statistical control. These factors may have introduced residual confounding, and the observed group differences should therefore not be interpreted as definitive causal effects. Future studies could use randomized assignment, baseline measures, or stronger covariate control where feasible.

Third, the sample remained limited in size, with 35 participants in each condition. Sensitivity analysis indicated that the achieved sample provided approximately 80% power to detect an omnibus effect of Cohen's *f* ≈ 0.31 (η^2^ ≈ 0.086) at α = 0.05, but was less sensitive to small effects. Larger samples would allow more precise effect estimation and further examination of participant-level differences.

Fourth, motivation and the cognitive-load components showed relatively low internal consistency. Cognitive load was therefore examined separately through intrinsic, extraneous, and germane components, and these findings were interpreted as exploratory. Future studies should further refine and validate measures of motivation and cognitive load in museum-learning contexts.

Fifth, objective knowledge performance was assessed immediately after the visit using a 10-item exhibition-based test. Because no pre-visit measure was administered, learning gains and baseline knowledge differences could not be assessed. In addition, full-sample item-level psychometric evaluation was unavailable; the test should therefore be interpreted as a study-specific, content-based measure rather than a standardized achievement instrument. Future research should incorporate pre-tests, validated measures, and delayed follow-up assessments to examine learning gains and longer-term retention.

Finally, the serious game was examined as an integrated visiting modality in which narrative, tasks, exhibit-based clues, spatial movement, feedback, and interface interaction occurred together. The study therefore cannot isolate the contribution of individual game components. Future research could manipulate specific design features to examine their distinct associations with visitor experience, cognitive-load components, and objective knowledge performance.

## 5 Conclusion

This study compared natural visiting, audio guidance, and a location-based serious game in an authentic museum setting, with attention to visitor experience, motivation, cognitive-load components, and objective learning outcomes. The results indicate that the location-based serious game condition was associated with stronger visitor experience and higher objective knowledge-test performance than the other two visiting modalities. These advantages were most evident in engagement, perceived knowledge/learning, meaningful experience, and emotional connection. At the same time, the findings show that the serious game did not uniformly outperform the other modalities across all dimensions. Its motivational advantage was limited, while intrinsic, extraneous, and germane cognitive load showed distinct patterns across conditions.

The study contributes to museum learning and cultural heritage serious game research by showing that the value of location-based serious games lies not simply in adding game elements to an exhibition, but in organizing museum visiting as a structured process of observation, interpretation, movement, and task-based engagement. When narrative, spatial progression, exhibit-based clues, and feedback are closely connected to exhibition content, serious games may support more active and meaningful forms of informal learning.

These findings also point to a central design challenge. Location-based serious games should guide visitors toward closer engagement with artifacts while avoiding unnecessary cognitive burden. Effective museum game design therefore requires a balance between narrative structure, task challenge, spatial interaction, and interpretive clarity. Rather than replacing existing forms of museum interpretation, location-based serious games may serve as a complementary modality for visitors and institutions seeking more participatory, situated, and learning-oriented museum experiences.

## Data Availability

The raw data supporting the findings can be made available by the authors upon reasonable request.
