# Supplementary material for: A location-based serious game in museum learning: a comparative field study of visitor experience, motivation, cognitive load, and objective learning outcomes
Source: Front Psychol. 2026 Oct 30;17:1979842. doi: 10.3389/fpsyg.2026.1979842 (PMC13760570; doi:10.3389/fpsyg.2026.1979842)
Supplement: Supplementary file 1 [file Supplementary_file_1.docx]

Supplementary Material

Note. The original questionnaire and knowledge-test materials were administered in Chinese. English translations are provided here for publication and review purposes. No participant responses, scores, signatures, or personal information are included in this supplementary file.

# 1 Supplementary Data

This supplementary file provides the materials used for the post-visit questionnaire, the exhibition-based objective knowledge test, and the semi-structured interviews. Questionnaire items were rated on a five-point Likert scale, where 1 = strongly disagree and 5 = strongly agree. The response scale is described here and is not repeated for each item in the tables.

# 2 Supplementary Tables

**Supplementary Table 1. Post-visit questionnaire items**

*Note. The table lists questionnaire items only; rating columns and participant responses are omitted. For analysis, the six motivation items were retained as a composite measure because the two three-item subsets showed limited internal consistency. Cognitive-load items were analyzed separately as intrinsic, extraneous, and germane components rather than as a single overall score.*

| **Construct** | **Sub-dimension** | **Item code** | **Item** |
| --- | --- | --- | --- |
| Cognitive load | Intrinsic cognitive load | ICL1 | During the visit, the content and tasks required me to process many pieces of information at the same time. |
| Cognitive load | Intrinsic cognitive load | ICL2 | The information during the visit was complex and difficult for me to understand. |
| Cognitive load | Extraneous cognitive load | ECL1 | During the visit, identifying important information required effort. |
| Cognitive load | Extraneous cognitive load | ECL2 | The design of the visit (game, audio guide, or museum visit) was not conducive to my effective learning. |
| Cognitive load | Extraneous cognitive load | ECL3 | During the visit, it was difficult for me to identify and connect key information. |
| Cognitive load | Germane cognitive load | GCL1 | During the visit, I worked hard to understand the relationship between details and the overall background information. |
| Cognitive load | Germane cognitive load | GCL2 | During the visit, I focused on correctly understanding all content. |
| Cognitive load | Germane cognitive load | GCL3 | The elements in the exhibition helped me better understand the learning content. |
| Motivation | Intrinsic motivation | IM1 | During the visit, I preferred challenging content because it allowed me to learn new things. |
| Motivation | Intrinsic motivation | IM2 | During the visit, I preferred content that aroused my curiosity, even though it was difficult. |
| Motivation | Intrinsic motivation | IM3 | Whenever possible, I would choose content that allowed me to learn and improve, even if it extended the visiting time. |
| Motivation | Extrinsic motivation | EM1 | Through this visit, being able to complete the visiting content smoothly was the most satisfying thing for me. |
| Motivation | Extrinsic motivation | EM2 | If possible, through this visit I wanted to gain more knowledge than most other visitors. |
| Motivation | Extrinsic motivation | EM3 | I hoped to gain more knowledge because showing what I learned to family, friends, or others was important. |
| Perceived knowledge/  learning | — | PKL1 | During this visit, I thought the exhibit information provided was clear. |
| Perceived knowledge/  learning | — | PKL2 | During this visit, I could understand most of what I saw and did in the exhibition. |
| Perceived knowledge/  learning | — | PKL3 | During this visit, I liked the graphics related to the exhibition. |
| Perceived knowledge/  learning | — | PKL4 | This visit enriched my knowledge and understanding of specific exhibits. |
| Perceived knowledge/  learning | — | PKL5 | During this visit, I discovered new information from the exhibits. |
| Engagement | — | ENG1 | During this exhibition visit, I felt enjoyment. |
| Engagement | — | ENG2 | I felt that I participated in this exhibition. |
| Engagement | — | ENG3 | I thought this exhibition visit was interesting. |
| Engagement | — | ENG4 | I felt that I was experiencing the exhibition, not just visiting it. |
| Engagement | — | ENG5 | This visit inspired me. |
| Meaningful experience | — | ME1 | During the visit, I was able to think about the meaning of the exhibits and their underlying implications. |
| Meaningful experience | — | ME2 | During the visit, I spent a lot of time thinking about the exhibition. |
| Meaningful experience | — | ME3 | During the visit, seeing precious and rare exhibits made me curious about the exhibition. |
| Meaningful experience | — | ME4 | After the visit, I was still interested in the exhibition theme and wanted to learn more. |
| Meaningful experience | — | ME5 | Seeing important exhibits in the exhibition was the most satisfying part of my visit. |
| Emotional connection | — | EC1 | Through this visit, I recalled the past. |
| Emotional connection | — | EC2 | During this visit, my feelings were stronger than those I usually experience in everyday life. |
| Emotional connection | — | EC3 | During this visit, I was impressed by the beauty of the exhibits. |
| Emotional connection | — | EC4 | Through this visit, I wanted to have objects like the exhibits I saw. |
| Emotional connection | — | EC5 | Through this visit, I felt connected with the exhibits. |

**Supplementary Table 2. Exhibition-based objective knowledge-test items and answer key**

*Note. The 10-item exhibition-based knowledge test was scored 1 point per correct response (total score: 0–10) and was used as a study-specific, content-based measure rather than a standardized achievement instrument. Because complete item-level responses were unavailable for the full sample, full-sample psychometric indices could not be retrospectively estimated.*

| **Content area** | **Item** | **Question** | **Response options** | **Correct answer** |
| --- | --- | --- | --- | --- |
| Pre-Qin period | 1 | Around 4,500 years before present, the Chengdu Plain had already developed settlement centers with centralized political authority. The earliest and largest ancient city on the Chengdu Plain was ____. | A. Baodun Ancient City B. Mangcheng, Dujiangyan C. Gaoshan Ancient City D. Shuanghe Ancient City | A |
| Pre-Qin period | 2 | The ____ site became the new political center of the Ancient Shu polity after Sanxingdui. It inherited the Sanxingdui cultural tradition and represented a flourishing stage of the Ancient Shu civilization. | A. Baodun B. Jinsha C. Liangzhu D. Xianrendong | B |
| Pre-Qin period | 3 | The Stone Rhinoceros has a full, sturdy body and short, thick limbs. Its primary function was ____. | A. Warding off evil and misfortune B. Guarding tombs C. Serving as a ritual vessel D. Controlling floods | D |
| Pre-Qin period | 4 | In late Shu culture, the “Boat for Carrying Souls” was used for ____. | A. Storage B. Transporting goods C. Funerary use D. River crossing | C |
| Pre-Qin period | 5 | In the Dujiangyan Irrigation System, Baopingkou, or the Bottle-Neck Channel, served the function of ____. | A. Diverting water and regulating water volume B. Discharging floods and flushing sediment C. Dividing water flow and sediment D. Stabilizing the water source | A |
| Han, Wei, Jin, and Northern and Southern Dynasties | 6 | During the Qin and Han periods, storytelling and performance arts were popular. Eastern Han pottery figurines unearthed in Chengdu vividly depict the exaggerated facial expressions and bodily postures of folk performers. In ancient China, such performers who were skilled in verbal performance and participated in song-and-dance activities were known as ____. | A. Lingren (伶人) B. Youling (优伶) C. Paiyou (俳优) D. Youren (优人) | C |
| Han, Wei, Jin, and Northern and Southern Dynasties | 7 | The Eastern Han ____ contains important information about local administration, education, and culture at the time. Inscriptions such as “the majestic Great Han” and “ranked among the Five Metropolises” reflect Chengdu’s important status during the Han dynasty. | A. Jingyun Stele B. Cao Quan Stele C. Pei Jun Stele D. Li Jun Stele | C |
| Han, Wei, Jin, and Northern and Southern Dynasties | 8 | The red and white meridian lines and acupoints on the lacquered meridian figure were mainly used to ____. | A. Showing the skeletal structure of the human body B. Precisely guiding acupuncture treatment C. Depicting the distribution of blood vessels D. Predicting the development of diseases | B |
| Han, Wei, Jin, and Northern and Southern Dynasties | 9 | In the late Western Jin period, the ethnic minority leaders Li Te and Li Xiong rose against Western Jin rule and established the ____ regime in Chengdu, one of the earliest regimes of the Sixteen Kingdoms period. | A. Eastern Jin B. Shu Han C. Cheng-Han D. Eastern Han | C |
| Han, Wei, Jin, and Northern and Southern Dynasties | 10 | The Henan Route of the Silk Road was an important route connecting the Southern Dynasties and the Western Regions. The starting point of this route was ____. | A. Dunhuang Commandery B. Tuyuhun City C. Yizhou, Chengdu D. Minzhou | C |

**Supplementary Table 3. Semi-structured interview guide**

*Note. The interviews were used as contextual and interpretive evidence. Questions were asked flexibly according to participants’ responses and the visiting condition they experienced.*

| **Interview focus** | **Guiding question** |
| --- | --- |
| Overall visiting experience | How would you describe your overall experience during this museum visit? |
| Engagement | Which parts of the visit made you feel most involved or interested? |
| Exhibit observation | Did the visiting activity help you pay attention to particular exhibits or exhibit details? Please explain. |
| Task comprehension | For participants in the serious game condition, how did you understand the game tasks, clues, and prompts? |
| Perceived learning | What new information or understanding did you gain from the exhibition during this visit? |
| Motivation | What encouraged you to continue the visit or complete the visiting activity? |
| Cognitive effort | Which parts of the visit required more attention, effort, or mental processing? |
| Narrative and spatial interaction | For participants in the serious game condition, how did the storyline, route, location-based interaction, and feedback influence your visit? |
| Emotional response | Did any exhibit, story, or task create a strong emotional response or personal connection? Please explain. |
| Comparison and reflection | Compared with ordinary viewing or listening-based interpretation, what was different about this visiting experience? |
| Suggestions | What improvements would you suggest for this visiting activity or serious game design? |
| Closing question | Is there anything else you would like to add about your museum visit? |

**Supplementary Table 4. Representative anonymized interview excerpts**

*Note. Excerpts were translated from anonymized Chinese interview summaries. The qualitative analysis was based on expanded interview summaries rather than verbatim transcripts. Participant codes contain no demographic information, and only representative excerpts are presented to protect confidentiality.*

| **Theme and participant code** | **Representative translated summary excerpt** | **Related interpretation** |
| --- | --- | --- |
| Narrative-guided exploration SG-P02 | “The background story placed me in the atmosphere of protecting cultural relics. Dialogues with artifacts and characters helped me understand the museum and history more deeply, and the game map guided me through different areas of the museum.” | The storyline, characters, and map helped organize route following and exhibit attention. |
| Exhibit-based reasoning SG-P03 | “The game content was not only about the artifacts themselves. It also connected the artifacts with wall diagrams and explanations, which helped visitors understand the historical background of the artifacts more deeply.” | Game tasks encouraged visitors to connect artifacts with contextual information and supported closer exhibit-based reasoning. |
| Emotional connection with artifacts SG-P02 | “The dialogues with many artifact characters made me feel the local characteristics of Chengdu and some ancient stories. I imagined scenes of ancient Chengdu, which made the visit more interesting than my usual museum visits.” | Character dialogue and localized narratives supported affective and imaginative responses to artifacts and cultural stories. |
| Increased task demand SG-P05 | “When I concentrated on solving puzzles, I had less attention for observing the details of other artifacts. Some puzzles were a little difficult for me, and it would be better if the game time were slightly shorter.” | Puzzle solving increased task-related effort and could compete with attention to other exhibits. |
| Active visiting experience SG-P01 | “If I had visited freely, I might only have browsed the introductions briefly and would not have remembered much. The game made me take on a role, follow the story, actively search for clues, and solve puzzles.” | The serious game shifted the visit from passive browsing to active exploration. |
